# Evaluating an Abbreviated Internet-Delivered Stress Recovery Intervention for Health Care Workers: Pre-Post Feasibility Study of Outcomes, Usability, and Acceptability

**DOI:** 10.2196/89312

**Published:** 2026-06-05

**Authors:** Austeja Dumarkaite, Anton Käll, Ramune Serapine, Evaldas Kazlauskas

**Affiliations:** 1 Center for Psychotraumatology, Institute of Psychology Faculty of Philosophy Vilnius University Vilnius Lithuania; 2 Center for Social and Affective Neuroscience Linköping University Linköping Sweden

**Keywords:** health care workers, internet-delivered intervention, stress recovery, abbreviated version, feasibility study

## Abstract

**Background:**

Health care workers face numerous occupational stressors that place them at heightened risk for burnout and poor mental health. Internet-delivered interventions have shown promise in reducing stress and related symptoms, yet adherence is often low, and users do not complete programs. Abbreviated interventions may help address engagement barriers such as high workload, limited time, and varying user preferences. There is a need to evaluate brief, accessible formats of internet-delivered programs for this population.

**Objective:**

This study aimed to examine the initial outcomes, usability, and acceptability of a 4-week abbreviated internet-delivered stress recovery intervention for health care workers. Specifically, it evaluated changes in stress recovery, perceived stress, depression and anxiety symptoms, and psychological well-being. The study also sought to understand participants’ experiences with the brief format to determine whether it meets their needs.

**Methods:**

This single-arm pre-post study examined a 4-week abbreviated version of the online guided cognitive behavioral therapy-based stress recovery program FOREST among self-enrolled health care workers recruited through professional networks (N=52; mean age 39.31, SD 11.31 years; 49/52, 94.2% women). Outcomes included stress recovery (the Recovery Experience Questionnaire), perceived stress (the Perceived Stress Scale-4), depression and anxiety symptoms (the Patient Health Questionnaire-4), psychological well-being (the World Health Organization Well-being Index), and usability and acceptability ratings.

**Results:**

We found that after the abbreviated version of the FOREST intervention participants showed moderate improvements in stress recovery (*d*=0.54, 95% CI 0.25-0.83); reductions in stress (*d*=–0.43, 95% CI –0.72 to –0.14), anxiety and depression symptoms (*d*=–0.51, 95% CI –0.80 to –0.22); and increase in psychological well-being (*d*=0.39, 95% CI 0.08-0.70). The majority (37/52, 71.2%) accessed all 6 modules. Users reported high satisfaction with the abbreviated program.

**Conclusions:**

While preliminary and limited by the pre-post design, these findings indicate that abbreviated internet-based stress recovery programs are a promising and practical tool for supporting the mental health of health care workers. Future research should examine the long-term effects, compare the abbreviated and standard versions, and explore implementation in routine practice.

## Introduction

Health care workers often face a range of stressors in their profession, which can contribute to psychological challenges and mental health issues. Common job-related stressors include high workload, poor working conditions, lack of positive feedback, and inequity in social interactions, among others [1-3]. In addition, many encounter morally injurious events such as emotional abuse by senior colleagues, patient physical abuse, and witnessing intentional harm, to name several [4]. The list of moral stressors can grow under specific circumstances; for instance, during the pandemic, health care workers frequently reported excessive patient care demands, inadequate resources, and a belief that administrators were not addressing critical care issues [5]. Unsurprisingly, in such a context, health care workers experience burnout [6] that remains elevated compared with prepandemic levels [7], high levels of stress, depression, and anxiety [8], insomnia [9], moral injury [10], risk for posttraumatic stress disorders [4], and suicide risk [3], among other things. These stressors can not only be linked to poor mental health outcomes but may also contribute to career change ideation [8]. Having in mind these difficulties experienced by health care workers, there is a clear and pressing need for mental health interventions.

In this context, on the individual level (while also recognizing necessary changes within the system), internet-delivered interventions can be highly valuable, as they offer benefits such as anonymity and the flexibility to access support at any time and from anywhere. A number of randomized controlled trials (RCTs) have been conducted recently in the health care workers’ samples, aiming to assess the effects of online interventions with various theoretical backgrounds, levels of guidance, lengths, among other features, overall showing encouraging results. Across these RCTs, it was demonstrated that such interventions can help to reduce work-related ruminations [11], specific types of burnout [12], stress [13-16], depression symptoms [16-18], anxiety symptoms [13,15-18], posttraumatic stress disorder symptoms [11]; foster psychological well-being [16,18], resilience [14], stress recovery [16], work-life integration, happiness, emotional thriving, and recovery [19]. However, the expected effects are not always observed, for instance, some studies report no changes in stress levels after such interventions [11,20], or the effects are not sustained for a longer period on specific symptoms [16].

In addition to evaluating the effects of internet-delivered interventions, their usability and acceptability represent important, yet often underreported, aspects. Studies that do report on adherence frequently find that participants do not engage with the full extent of the intervention. For instance, it was reported that participants used only about 3 out of 6 sessions [20], watched less than half of the provided video content [11], or showed a nearly 50% drop in sign-ins from the first to the last intervention module [16]. However, in shorter interventions, such as a 4-week program, adherence appeared more favorable, with participants interacting with the intervention more than once per day on average [18]. Regarding acceptability, most participants reported that they liked the interventions, found them useful, and easy to use [11,16,20]. Nevertheless, they did not necessarily find them easy to implement in daily life [20]. Qualitative data suggest that health care workers who used internet-delivered interventions felt supported and cared for, valued the resources provided, and appreciated the opportunity to reflect on or express their feelings; at the same time, they offered suggestions for improving future interventions [21]. Health care workers who discontinued their use of an internet-delivered program named therapist support, number of reminders, content-related aspects—engagement, relevance, format, complexity—among other things, as either possible barriers or facilitators to use the intervention [22]. Thus, while online interventions are generally well appreciated, they are frequently not fully used. Also, it is apparent that they do not necessarily correspond to the expectations of their users and that can stand as an important barrier.

Given the demanding working conditions of health care workers, as well as both the opportunities and limitations of internet-delivered interventions for this population, there is a need to explore diverse approaches to providing support. While ongoing research continues to investigate factors such as the type of guidance and the role of reminders, other important aspects remain underexplored. One such aspect is the length of the intervention, which may be particularly critical for specific intervention delivery contexts and populations. For example, high workload—frequently cited by health care workers as a major source of stress [2,3] —can act as a significant barrier to accessing and engaging with psychological support. In this context, an abbreviated version of an internet-delivered intervention could be a simple yet valuable alternative. Moreover, the stigma associated with seeking psychological help among health care professionals might be lessened by offering a brief, accessible tool aimed at stress management. In terms of interventions, evidence suggests that many participants do not complete full-length interventions [11,16,20,23], raising the question of whether a briefer in time format might encourage greater engagement and completion. If participants are more likely to complete a briefer intervention, they may also be more likely to receive the full intended therapeutic dose, potentially enhancing outcomes. Additionally, qualitative findings indicate that individuals vary in their interest and preferences regarding the type of support offered [22]. This further highlights the importance of developing and evaluating a range of alternative formats to better meet the diverse needs of health care workers.

Hence, in this study, we aimed to explore the initial outcomes, usability, and acceptability of an abbreviated in time online stress recovery intervention for health care workers using a single-arm pre-post design. Although multiple RCTs have demonstrated the efficacy of online mental health interventions for health care workers, many of these programs require several weeks of engagement. Health care workers frequently report significant time constraints and workload demands that may limit sustained participation in longer interventions. Previously, a 6-week version of the intervention demonstrated positive outcomes and high satisfaction among health care workers in 2 RCTs; at the same time, a substantial proportion of participants have not completed the full program [16,23]. In addition, its duration may reduce accessibility and scalability in high-demand clinical settings. On that account, this study sought to evaluate the changes in health care workers’ stress recovery (primary outcome), perceived stress, symptoms of depression and anxiety, and psychological well-being (secondary outcomes) after the same online cognitive behavioral therapy-based stress recovery intervention delivered in 4 weeks. In addition, we aimed to assess health care workers’ evaluation of such an abbreviated in time format of the intervention to better understand its usability and acceptability. In our research, we follow the UK Medical Research Council’s guidance for developing and evaluating interventions [24]. We consider this study to be in a feasibility phase, which will be followed by an evaluation study and subsequently an implementation study in specific health care settings.

## Methods

### Procedure

A single-arm pre-post intervention design study was conducted, aimed at evaluating the initial outcomes, usability, and acceptability of an abbreviated in time (from 6 to 4 weeks) online stress recovery intervention for health care workers, FOREST. Assessments took place at two time points: pretest (October-November 2023) and posttest (December 2023). Self-report data were collected via a secure internet-intervention delivery platform, Stillminder, developed at the Center for Psychotraumatology at Vilnius University. This study is reported in accordance with the TREND (Transparent Reporting of Evaluations with Nonrandomized Designs) statement for nonrandomized studies [25]. The completed checklist is provided in Multimedia Appendix 1.

The recruitment process took place in October-November 2023 (date of first recruitment: October 31, 2023) in Lithuania. Participants were recruited following a post on the Center for Psychotraumatology at Vilnius University Facebook page and reposting it to several largest professional health care networks. Individuals interested in taking part in the study were directed to register on the study’s official website, where comprehensive information about the program was made available.

**Figure 1:**
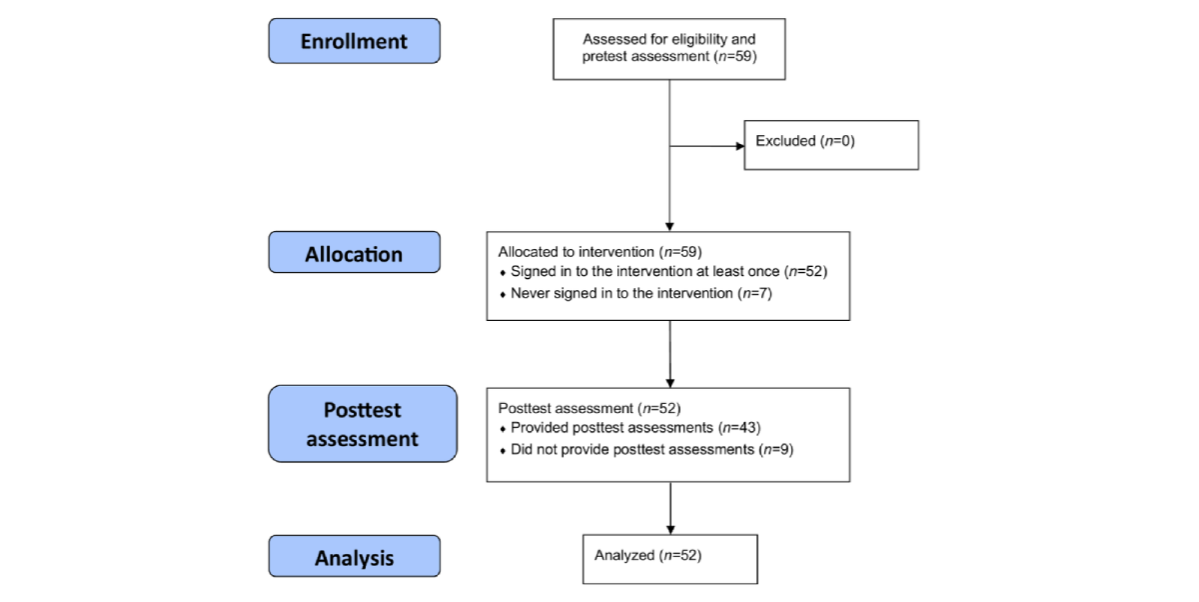
Flowchart of the study.

Informed consent was obtained electronically, and participants who agreed to participate in the study could further complete baseline assessment questionnaires online. Those who completed the pretest assessments in full were subsequently contacted via telephone for a brief screening interview to confirm their eligibility. During this call, participants were also given an opportunity to ask questions about the program and clarify any procedural details. A schematic representation of the participant flow throughout the study is provided in Figure 1.

Eligibility criteria included being a worker in a health care setting, being at least 18 years old, having proficiency in the Lithuanian language, and having access to an internet-enabled device. Exclusion criteria were defined as the presence of an acute psychiatric crisis, elevated risk of suicide, substance use addiction (alcohol or drugs), or involvement in interpersonal violence. No participants were excluded after the eligibility calls due to not meeting the inclusion criteria or meeting the exclusion criteria.

In addition to the eligibility call, participants were contacted by study researchers on 2 more times—in the middle of the intervention and immediately after the intervention. The aim of the call was to encourage participants to continue with the intervention, ask about the initial feedback of their experience, and answer questions related to the intervention and study.

### Participants

Overall, 59 individuals registered for the study, provided informed consent, completed the baseline questionnaires, and after the eligibility assessment via a call were included in the study. 7 participants never accessed any of the 6 modules in the program; therefore, they were excluded from the analysis, and the final sample included 52 participants. As this is a feasibility study, the sample size is not based on a formal sample size calculation but on practical and logistical considerations. All baseline descriptive data for the study participants are presented in Table 1.

Participants also reported experiencing various stressors over the past 2 years that they perceived as a significant burden at the time of the baseline assessment. The most frequently cited stressors included having too much or too little work (45/52, 86.5%), pressure to meet deadlines or time constraints (29/52, 55.8%), family conflicts (23/52, 44.2%), financial difficulties (23/52, 44.2%), and workplace conflicts (22/52, 42.3%). All stressors experienced by participants at baseline are presented in Table 2.

**Table 1:** Characteristics of study participants (N=52) at baseline.

Variables			Values
**Gender, n (%)**			
	Women	49 (94.2)	
	Men	3 (5.8)	
Age (years), mean (SD); range			39.31 (11.31); 24-61
**Education, n (%)**			
	Secondary or lower	1 (1.9)	
	Professional college	12 (23.1)	
	University degree	39 (75)	
**License category, n (%)**			
	Doctor	16 (30.8)	
	Nurse	20 (38.5)	
	Other	11 (21.1)	
	No license	5 (9.6)	
**Employment status, n (%)**			
	<full-time	3 (5.8)	
	Full-time	23 (44.2)	
	>full-time	26 (50)	
**Type of services^a^** **, n**			
	Outpatient care	34	
	Inpatient care	19	
	Rehabilitation	2	
	Nursing	2	
	Emergency	4	
	Intensive care	4	
	Other	6	
**Work experience, n (%)**			
	<2 years	6 (11.5)	
	2-5 years	11 (21.2)	
	6-10 years	12 (23.1)	
	>10 years	23 (44.2)	
**Residence of work placement^a^** **, n**			
	Large and medium-sized cities	51	
	Small towns and villages	5	
**Subjective family income, n (%)**			
	<average	4 (7.7)	
	Average	38 (73.1)	
	>average	10 (19.2)	
**Long-term relationship, n (%)**			
	No	15 (28.8)	
	Yes	37 (71.2)	
**Current use of psychologist or psychotherapist services, n (%)**			
	No	40 (76.9)	
	Yes	12 (23.1)	
**Recent use of other self-help app, n (%)**			
	No	40 (76.9)	
	Yes	12 (23.1)	
**Taking medication due to mental health difficulties, n (%)**			
	No	45 (86.5)	
	Yes	7 (13.5)	
**Taking medication to improve well-being, n (%)**			
	No	31 (59.6)	
	Yes	21 (40.4)	
**Alcohol consumption to improve well-being, n (%)**			
	No	35 (67.3)	
	Yes	17 (32.7)	
**Psychoactive substances consumption to improve well-being, n (%)**			
	No	50 (96.2)	
	Yes	2 (3.8)	
**Suicidal thoughts, n (%)**			
	No	45 (86.5)	
	Yes	7 (13.5)	

^a^Participants could choose more than one answer from the list.

**Table 2:** Stressors experienced by participants (N=52) at baseline.

Stressors		Values	
Divorce or separation, n (%)		11 (21.2)	
Family conflicts, n (%)		23 (44.2)	
Conflicts in work life, n (%)		22 (42.3)	
Conflicts with neighbors, n (%)		7 (13.5)	
Illness of a loved one, n (%)		17 (32.7)	
Death of a loved one, n (%)		10 (19.2)	
Adjustment to retirement, n (%)		0 (0)	
Unemployment		4 (7.7)	
Too much or too little work		45 (86.5)	
Pressure to meet deadlines or time pressure		29 (55.8)	
Moving to a new home		19 (36.5)	
Financial problems		23 (44.2)	
Own serious illness		6 (11.5)	
Serious accident		2 (3.8)	
Assault		3 (5.8)	
Termination of an important leisure activity		15 (28.8)	
Other stressful events		10 (19.2)	
Overall, mean (SD); range			4.73 (2.34); 0-9

### Completers and Dropouts Comparison

To examine potential attrition bias, baseline differences between participants who completed the posttest assessment (n=43) and those who dropped out (n=9) were analyzed. There were no significant differences in demographic characteristics at pretest, including gender (*χ^2^*_1_=0.6; *P*=.45), age (*t*_13.13_=–1.95; *P*=.07), education (*χ^2^*_1_=1.5; *P*=.22), employment status (*χ^2^*_1_=2.8; *P*=.09), work experience (*χ^2^*_1_=1.9; *P*=.16), family income (*χ^2^*_1_=0.0; *P*=.99), or partnership status (*χ^2^*_1_=0.2; *P*=.20). Completers and dropouts also did not significantly differ on any baseline outcome measures. No differences were observed for stress recovery (*t*_11.87_=–0.46; *P*=.66), perceived stress (*t*_11.45_=–0.96; *P*=.36), depression and anxiety symptoms (*t*_10.59_=1.42; *P*=.19), or psychological well-being (*t*_17.18_=–1.91; *P*=.07).

### Intervention

The intervention FOREST, previously detailed by Jovarauskaite et al [26], is an internet-based program grounded in cognitive behavioral therapy and incorporates elements of mindfulness. The intervention was designed by clinical psychologists and researchers with expertise in stress-related disorders and digital mental health interventions. The intervention comprises 6 modules: Introduction, Psychological detachment, Distancing, Mastery, Control, and Keeping the change alive. Each module includes psychoeducational content specific to the module’s theme, a series of practical exercises that are reviewed and commented on by a trained psychologist once completed and prompts to contact a psychologist. The intervention was delivered via a secure internet-intervention delivery platform, Stillminder*.*

Originally, the intervention was developed as a 6-week program, where participants received access to a new module every week on a designated day, accompanied by an email notification. For this study, the intervention delivery period was shortened to 4 weeks: in week one participants received access to the Introduction, week two – Psychological detachment and Distancing, week three – Mastery and Control, week four – Keeping the change alive. Additional weekly reminders were sent to participants who had not logged into the platform, accessed new content, or completed the assigned exercises. Importantly, no changes were made to the content, and no modules were omitted.

A total of 27 students in the Clinical psychology Masters’ program were involved as psychologists in the delivery of the intervention. Prior to providing support, they received training conducted by a clinical psychologist (AD) to ensure familiarity with the intervention protocol and feedback guidelines. Their responsibilities included providing individualized feedback on completed exercises, responding to participants’ inquiries, and offering psychological support. Participants were guaranteed a response within 24 hours. All psychologist responses were guided by standardized protocols, and weekly supervision meetings throughout the intervention period were held in 2 groups to ensure adherence to the intervention framework, discuss participants’ situations, and possible feedback. Additionally, messages written by the psychologists were reviewed by a clinical psychologist (AD) until they consistently met the established guidelines.

### Measures

#### Stress Recovery

The Recovery Experience Questionnaire (REQ) [27] was used to assess stress recovery. This instrument consists of 16 items measuring four components of stress recovery: (1) psychological detachment (eg, “I forget about work”), (2) relaxation (eg, “I kick back and relax”), (3) mastery (eg, “I learn new things”), and (4) control (eg, “I feel like I can decide for myself what to do”) with 4 items on each subscale. Participants rated each item using a 5-point Likert scale ranging from 1 (“totally disagree”) to 5 (“totally agree”). In this study, the internal consistency of the REQ at baseline was excellent, with a Cronbach α of 0.90. Each subscale also showed good to excellent internal consistency: psychological detachment (α=0.89), relaxation (α=0.91), mastery (α=0.81), and control (α=0.85). We report the sum scores of all 16 items, as well as the sum scores of all 4 subscales.

#### Stress

The Perceived Stress Scale-4 (PSS-4) [28] was used to assess participants’ perceived stress levels over the past month. This instrument consists of 4 questions (eg, “In the last month, how often have you felt that you were unable to control the important things in your life?”). Participants rated each item using a 5-point Likert scale ranging from 0 (“never”) to 4 (“very often”). In this study, the internal consistency of the PSS-4 at baseline was acceptable, with a Cronbach α of 0.75.

#### Symptoms of Depression and Anxiety

The Patient Health Questionnaire-4 (PHQ-4) [29] was used to assess participants’ symptoms of depression and anxiety over the past 2 weeks. This instrument consists of 4 items and 2 subscales with 2 items each: symptoms of depression (eg, “Little interest or pleasure in doing things”), and symptoms of anxiety (eg, “Feeling nervous, anxious or on edge”). Participants rated each item using a 4-point Likert scale ranging from 0 (“not at all”) to 3 (“nearly every day”). In this study, the internal consistency of the PHQ-4 at baseline was good, with a Cronbach α of 0.84. Each subscale also showed acceptable to good internal consistency: depression (α=0.81) and anxiety (α=0.71).

In addition to treating the PHQ-4 total score as a continuous variable, we also used established cut-off scores to classify participants into levels of overall distress, defined as the combined burden of depressive and anxiety symptoms. Consistent with the cut-off scores proposed by Kroenke et al [29], total PHQ-4 scores were interpreted as follows: 0-2 (none; low risk), 3-5 (mild distress), 6-8 (moderate distress), and 9-12 (severe distress). For the purposes of this study, participants were further grouped into two categories: none to mild distress (scores 0-5) and moderate to severe distress (scores 6-12).

#### Psychological Well-Being

The World Health Organization Well-being Index (WHO-5) [30] was used to assess participants’ psychological well being over the past 2 weeks. This instrument consists of 5 items (eg, “I have felt cheerful and in good spirits”). Participants rated each item using a 6-point Likert scale ranging from 0 (“at no time”) to 5 (“all of the time”). In this study, the internal consistency of the WHO-5 at baseline was good, with a Cronbach α of 0.83.

#### Usability and Acceptability of the Intervention

Participants were asked to assess the usability and acceptability of the intervention by rating its usefulness (1=“not useful at all” to 5=“very useful”), overall satisfaction (1=“did not like it at all” to 5=“liked it a lot”), and ease of use (1=“not easy at all” to 5=“very easy”). Also, they were asked to rate the program content—texts, videos, and audios (0=“very negative” to 10=“very positive”). Additionally, participants were asked to provide subjective evaluations of the intervention’s impact on their mental well-being and physical health (1=“worsened a lot” to 5=“improved a lot”) and their general self-awareness and understanding of well-being (1=“not at all” to 5=“definitely improved”). Finally, participants were asked to indicate the likelihood of recommending the program to others (1=“not at all” to 5=“definitely would recommend”).

### Statistical Analysis

The statistical analyses were conducted using the software R (R Foundation for Statistical Computing) [31]. For all analyses, an α level of .05 was used as cut-off for statistical significance. CIs are described at 95%. For the model-implied outcomes, confidence intervals were estimated using the profile method from the *lme4* package [32]. The intention-to-treat principle was used, meaning that all participants that were included in the study provided data for the analyses. No data imputation was applied. Missing data were handled using maximum likelihood estimation under the missing at random assumption [33]. For analyzing treatment outcomes, we used a series of linear mixed effects models, specified using the *lme4* package [32]. Models were fitted with random intercepts, but we only estimated the fixed effect of slope due to having only 2 time points. The fixed effect of interest was the comparison between the pre- and posttreatment timepoints. The significance of the estimates from the fixed effects was tested using a Wald test. Restricted maximum likelihood was used for estimating model parameters. The models were estimated using the Satterwaite method for calculating degrees of freedom. Within-group effect sizes were calculated using the estimated marginal means and the estimated standard errors (both from the *emmeans* function).

### Ethical Considerations

The study was approved by Vilnius University Psychology Research Ethics Committee (Reference number 2021-03-22/61). All procedures followed were in accordance with the ethical standards of the responsible committee and with the World Medical Association Declaration of Helsinki. Prior to participants accessing the baseline questionnaires, informed consent was obtained. Participants were provided with clear and comprehensive information outlining the purpose of the study, the procedures involved, the voluntary nature of participation, and their right to withdraw at any time. Data were collected via a secure Stillminder software developed by Vilnius University and stored securely on a password-protected University computer, accessible only to authorized members of the research team. Participants were assured that only aggregated data would be reported in publications and presentations, and that no information that could identify them individually would be disclosed. Participants did not receive any form of financial or material compensation for their involvement in the study.

## Results

### Intervention Outcomes

#### Overview

**Figure 2:**
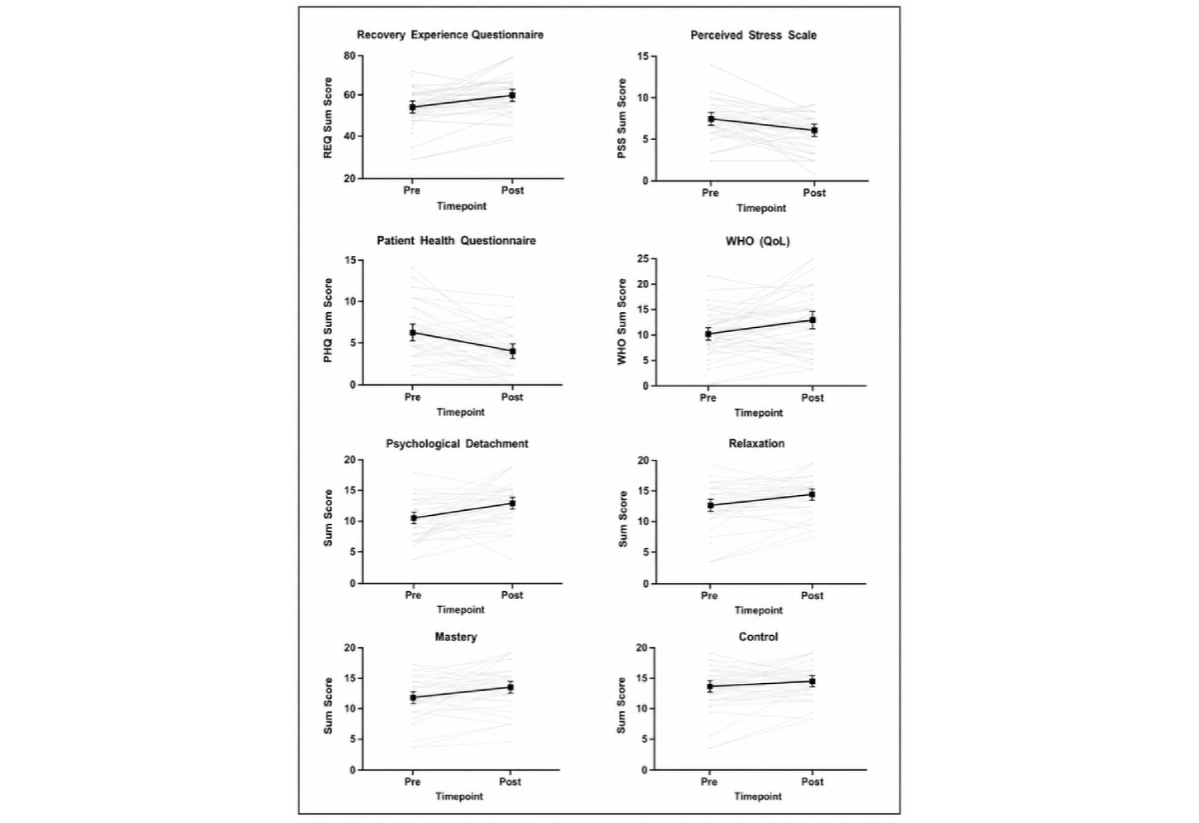
Observed sum scores for the outcomes with individual data points and 95% CIs for the observed means. PHQ-4: Patient Health Questionnaire, 4-item version; PSS-4: Perceived Stress Scale, 4-item version; QoL: quality of life; REQ: Recovery Experience Questionnaire; WHO-5: World Health Organization Well-Being Index.

The observed and estimated means, along with within-group effect sizes, are presented in Table 3. The change from pre- to posttreatment for REQ, PHQ, PSS, and WHO are visualized in Figure 2. The change in the different domains of the REQ measure is also visualized in Figure 2.

**Table 3:** Observed and estimated means for the outcome measures.

Measure	Pretreatment ratings			Posttreatment ratings (observed)			Pretreatment ratings (model implied marginal means)		Posttreatment ratings (model implied marginal means)		Within-group effect size for the estimated means
	Mean (SD)	n	Mean (SD)		n	Mean (SE)		Mean (SE)		Cohen *d* (95% CI)	
REQ^a^	50.39 (10.12)	52	57.91 (10.66)		43	50.4 (1.44)		57.8 (1.55)		0.54 (0.25-0.83)	
Psychological detachment	10.48 (3.53)	52	13.47 (3.42)		43	10.5 (0.48)		10.5 (0.48)		0.63 (0.33-0.92)	
Relaxation	13.21 (3.47)	52	14.93 (2.89)		43	13.2 (0.45)		14.9 (0.48)		0.42 (0.13-0.71)	
Mastery	12.25 (3.22)	52	14.28 (3.45)		43	12.2 (0.46)		14.2 (0.49)		0.47 (0.18-0.76)	
Control	14.19 (3.41)	52	15.23 (2.69)		43	14.2 (0.43)		15.2 (0.49)		0.23 (0.01-0.45)	
PSS-4^b^	7.90 (2.60)	52	6.37 (2.38)		43	7.9 (0.35)		6.41 (0.38)		–0.43 (–0.72 to –0.14)	
PHQ-4^c^	5.40 (2.92)	52	3.47 (2.35)		43	5.4 (0.37)		3.57 (0.41)		–0.51 (–0.80 to –0.22)	
WHO-5^d^	10.10 (4.32)	52	12.84 (5.88)		43	10.1 (0.70)		12.70 (0.76)		0.39 (0.08-0.70)	

^a^REQ: Recovery Experience Questionnaire.

^b^PSS-4: Perceived Stress Scale, 4-item version.

^c^PHQ-4: Patient Health Questionnaire, 4-item version.

^d^WHO-5: World Health Organization Well-Being Index.

#### REQ

The results for the linear mixed effects model suggested a significant increase in the overall scale during the treatment phase (*b*=5.23, SE=1.14; *P<*.001). Similarly, we found a significant increase for all of the subscales: psychological detachment (*b=*2.11, SE=0.45; *P<*.001); relaxation (*b*=1.19, SE=0.32; *P<*.001); mastery (*b*=1.38, SE=0.34; *P<*.001); control (*b*=5.23, SE=0.72; *P=*.03).

#### PSS-4

The linear mixed effects model suggested a significant decrease in stress during the treatment phase (*b*=–1.05, SE=0.32; *P=*.002).

#### PHQ-4

The linear mixed effect model suggested a significant decrease in symptoms of depression during the treatment phase (*b*=–1.30, SE=0.32; *P<*.001).

#### WHO-5

The linear mixed effects model implied a significant increase in quality of life from pre- to posttreatment (*b*=1.82, SE=0.57; *P=*.002).

### Change in Symptom Severity Categories

Given the exploratory nature and limited sample size of this feasibility study, statistical significance should be interpreted cautiously. Therefore, we additionally examined change across established symptom severity categories of the psychological distress measure. Among participants classified as having moderate to high distress at baseline and providing posttest data (n=18), 14 (77.8%) moved to none to mild distress levels, whereas 4 participants shifted from none to mild distress to moderate to high distress levels. A McNemar test indicated that this shift in symptom classification over time was statistically significant (*P*=.03). Overall, this pattern suggests a reduction in elevated distress symptoms over the study period.

### Use of the Intervention

Among the participants who accessed at least 1 of the 6 intervention modules (n=52), 23.1% (12/52) signed in fewer than 5 times, 21.2% (11/52) signed in 5-10 times, 34.6% (18/52) signed in 11-20 times, and 21.2% (11/52) signed in more than 20 times. Regarding time spent on the program each week, 40.5% (17/42) reported engaging for less than 15 minutes, another 40.5% (17/42) for 15-30 minutes, and 19% (8/42) for more than 30 minutes.

The majority of participants (71.2%, 37/52) accessed all 6 modules. Of the remaining participants, three accessed five modules, one accessed four, four accessed three, two accessed two, and five accessed only one module. Module-specific access rates were as follows: 98.1% (51/52) accessed the first module (Introduction), 90.4% (47/52) the second (Psychological Detachment), 84.6% (44/52) the third (Distancing), 78.8% (41/52) the fourth (Mastery), 75% (39/52) the fifth (Control), and 76.9% (40/52) the sixth (Keeping the Change Alive); 82.7% of participants (43/52) completed the posttest assessment, resulting in an attrition rate of 17.3% (9/52) at posttest.

### Acceptability of the Intervention

Among the participants who accessed at least 1 module of the program and completed the posttest assessment, including the evaluation of the intervention (n=42), the majority rated the program as useful (36/42, 85.7%), satisfactory (40/42, 95.2%), and easy to use (41/42, 97.6%). Program content, specifically texts, videos, and audios, was evaluated positively on a 10-point Likert scale, with mean scores of 8.81 (SD 1.81) for texts, 8.67 (SD 2.25) for videos, and 9.31 (SD 1.44) for audios. Furthermore, a substantial proportion of participants reported improvements in their well-being after the program: 66.7% (28/42) participants noted enhanced mental well-being, 50% (21/42) reported better physical health, and 76.2% (32/42) indicated a deeper understanding of themselves and their overall well-being. Additionally, most participants (85.7%, 36/42) stated that they would recommend the FOREST program to others. In terms of device use, 52.4% (22/42) of participants accessed the program via mobile phones, while 47.6% (20/42) used computers. No adverse events were observed during the intervention delivery time.

## Discussion

### Principal Findings

In this study, using a pre-posttest design, we investigated the changes over time in various psychological outcomes among health care workers who used an abbreviated 4-week internet-based stress recovery intervention. Specifically, we examined the changes in stress recovery, perceived stress, symptoms of anxiety and depression, and psychological well-being. Additionally, we evaluated the usability and acceptability of this abbreviated form of the intervention. The results indicated that after using FOREST stress recovery program, health care workers effectively enhanced stress recovery skills, including psychological detachment, relaxation, mastery, and control. They also showed reduced stress, symptoms of depression and anxiety, and improvement in psychological well-being. Furthermore, the usability and acceptability of the intervention were good, and participants reported a high level of overall satisfaction with the program.

Our study demonstrated preliminary signals of change in health care workers’ ability to recover from work-related stress after an abbreviated 4-week duration online stress recovery intervention. The specific group, which is frequently exposed to high workloads, time pressure, interpersonal conflicts at work and home, and financial strain, showed enhanced capacity to mentally and physically detach from work, engage in relaxation, pursue stimulating and distracting activities, explore new learning opportunities, and make more intentional decisions about how to spend their leisure time. Importantly, after using this intervention, health care workers not only improved their stress recovery skills but also reported greater confidence in handling personal challenges and a stronger sense of control over important aspects of their lives. They also felt less nervous and depressed, which is of extreme relevance given that health care workers are a group of people who face these difficulties a lot. Besides the alleviation of these difficult symptoms, health care workers could generally feel calmer, relaxed, cheerful, active, and interested in things that are relevant to them. Similar findings can be found in 2 earlier studies that explored the effects of the same 6-week duration stress recovery intervention in nurses and broader health care staff samples [16,23], suggesting that this abbreviated form of the intervention could also be applicable and stand next to the standard version when providing internet-delivered support. Across all 3 studies, small to moderate effects were observed on key outcomes, indicating consistent beneficial changes after using the program. Although effect sizes in this study were in some cases slightly smaller than those reported previously, they remained within the same overall range and demonstrate that the shortened format could retain meaningful clinical relevance. Moreover, the current results support broader trends observed in other intervention studies targeting health care workers, which have reported beneficial effects on stress, anxiety, and depression [13-15,17-19] as well as improvements in overall psychological well-being [18,19].

Regarding the usability of the 4-week online stress recovery program, most participating health care workers accessed all the modules; however, there was a decline in use of approximately 25% (13/52 for the fifth module and 12/52 for the sixth module) for the final modules. When compared to the standard 6-week version of the same program, adherence to the abbreviated version was somewhat higher in this study. In one RCT, slightly more than half of participants accessed the final module [16], while in another RCT, only half of the participants completed all 6 modules [23]. This pattern is consistent with findings from other internet-delivered interventions targeting health care workers, where participants often completed only around half of the available content [11,20]; except for a shorter 4-week intervention, which was used by health care workers more than once per day [18]. Although it is very preliminary and warrants more systematic evaluation, they suggest that shorter interventions may be more feasible for full completion, particularly in demanding health care settings.

In addition to its usability, health care workers evaluated the 4-week program positively in terms of ease of use, satisfaction, and perceived usefulness. More than half of the users felt that the program improved their well-being and helped to understand themselves better. Importantly, the majority of those who used this program would recommend it to colleagues facing similar challenges. These findings are consistent with evaluations of the original FOREST program in 2 RCTs, which also demonstrated high levels of user satisfaction [16,23], as well as with other online interventions aimed at health care workers [11,20]. Although more in-depth exploration regarding health care workers’ experiences of such an intervention would provide valuable insights, the current findings suggest that brief online stress recovery program is both feasible and well-received. Thus, such interventions may serve as an appealing and practical option for health care workers.

### Limitations and Future Directions

It is important to acknowledge several limitations of this study and outline key directions for future research to meaningfully advance this area of inquiry. First, as this was an initial study, a pre-post design was used. While useful for preliminary insights, this design offers only limited information about the effects and usability of the intervention, as it does not allow for comparison with a control group—either an active control condition or the standard-length version of the intervention, depending on the research objective. Moreover, without a control group, alternative explanations for the observed changes cannot be ruled out, including regression to mean, expectancy effects, or other nonspecific factors such as participants’ increased self-monitoring over time. Additionally, data were collected at only 2 time points, which limits the ability to draw conclusions about the long-term sustainability of the observed effects. The reliance on 2 measurement occasions also restricts the possibility of examining trajectories of change or distinguishing temporary fluctuations from stable improvements. In addition to that, abbreviated forms of measures to assess the changes in perceived stress and symptoms of anxiety and depression were used in this initial study, which may limit precision of the assessed constructs. Another notable limitation is the use of a self-referred sample, which may represent a specific subgroup of individuals already motivated to seek psychological support, open to low-intensity online formats, and potentially more ready for change than the general population of health care workers. Moreover, although module completion was high, this may have been influenced by the interactive format and follow-up support, which may not be feasible in scaled interventions. It is also important to note the limited generalizability of the findings, as the sample consisted predominantly of highly educated female participants working primarily in large and medium-sized cities. Consequently, the results may not generalize to male health care workers, those with different educational backgrounds, or professionals working in rural settings. Finally, potential bias related to attrition should be considered. Although the significance tests did not indicate any systematic differences between those who completed the posttreatment assessment and those who did not, we are unable to conclude whether dropout might relate to other variables, which could affect the generalizability of the findings, as dropout may have introduced systematic differences between those who completed the study and those who did not, thereby affecting the generalizability of the findings.

Looking ahead, several future research directions are warranted. It would be valuable to conduct a comparative study examining this abbreviated intervention against either the full-length version or an active control condition, to better understand its relative effects. Furthermore, it is essential to pursue implementation studies, exploring how such a program can be integrated into routine health care settings. We envision this online stress recovery intervention not as a clinical treatment, but rather as a mental health promotion tool—a brief, low-intensity program that health care workers can access individually, with minimal asynchronous support from a psychologist. In this context, economic evaluation also becomes relevant. Assessing the cost-effectiveness of such an intervention in health care settings could provide important insights into its potential value not only at the individual level but also within broader organizational and systemic frameworks.

### Conclusions

This study provides preliminary evidence that health care workers, after using an abbreviated 4-week internet-based stress recovery intervention FOREST, may improve stress recovery skills, reduce perceived stress and symptoms of anxiety and depression, and enhance psychological well-being. These findings suggest that an abbreviated format may retain meaningful effects and offer a practical alternative to a longer intervention format.

In addition, the 4-week program demonstrated good usability, with most health care workers completing the majority of modules, although engagement declined toward the end. Adherence was somewhat higher than in the full 6-week version, suggesting that an abbreviated intervention may be more feasible and better suited for completion in demanding health care settings.

Finally, health care workers evaluated the 4-week program positively in terms of ease of use, satisfaction, and usefulness, with many reporting improved well-being and willingness to recommend it to others. Overall, the findings suggest that brief online stress recovery interventions are feasible, well-received, and represent a practical support option for health care workers.

Moving forward, future research should use controlled and longitudinal designs, include more diverse samples, and focus on implementation and cost-effectiveness to better evaluate the intervention’s real-world applicability in health care settings.

## Appendix

Multimedia Appendix 1 TREND checklist.

## Abbreviations

PHQ-4: Patient Health Questionnaire-4
PSS-4: Perceived Stress Scale-4
RCT: randomized controlled trial
REQ: Recovery Experience Questionnaire
TREND: Transparent Reporting of Evaluations with Nonrandomized Designs
WHO-5: World Health Organization Well-being Index

## Data Availability

The datasets generated or analyzed during this study are available from the corresponding author on reasonable request.
